# Impaired Maintenance of Interpersonal Synchronization in Musical Improvisations of Patients with Borderline Personality Disorder

**DOI:** 10.3389/fpsyg.2017.00537

**Published:** 2017-04-27

**Authors:** Katrien Foubert, Tom Collins, Jos De Backer

**Affiliations:** ^1^Music Therapy, Department of Music, LUCA School of Arts, Association KULeuvenLeuven, Belgium; ^2^Department of Psychology, Lehigh UniversityBethlehem, PA, USA; ^3^Music Artificial Intelligence Algorithms, Inc.Davis, CA, USA

**Keywords:** interpersonal synchronization, musical improvisation, interpersonal functioning, borderline personality disorder, music information retrieval, music therapy, attachment, timing

## Abstract

Borderline personality disorder (BPD) is a serious and complex mental disorder with a lifetime prevalence of 5.9%, characterized by pervasive difficulties with emotion regulation, impulse control, and instability in interpersonal relationships and self-image. Impairments in interpersonal functioning have always been a prominent characteristic of BPD, indicating a need for research to identify the specific interpersonal processes that are problematic for diagnosed individuals. Previous research has concentrated on self-report questionnaires, unidirectional tests, and experimental paradigms wherein the exchange of social signals between individuals was not the focus. We propose joint musical improvisation as an alternative method to investigate interpersonal processes. Using a novel, carefully planned, ABA′ accompaniment paradigm, and taking into account the possible influences of mood, psychotropic medication, general attachment, and musical sophistication, we recorded piano improvisations of 16 BPD patients and 12 matched healthy controls. We hypothesized that the insecure attachment system associated with BPD would be activated in the joint improvisation and manifest in measures of timing behavior. Results indicated that a logistic regression model, built on differences in timing deviations, predicted diagnosis with 82% success. More specifically, over the course of the improvisation B section (freer improvisation), controls' timing deviations decreased (temporal synchrony became more precise) whereas that of the patients with BPD did not, confirming our hypothesis. These findings are in accordance with previous research, where BPD is characterized by difficulties in attachment relationships such as maintaining strong attachment with others, but it is novel to find empirical evidence of such issues in joint musical improvisation. We suggest further longitudinal research within the field of music therapy, to study how recovery of these timing habits are related to attachment experiences and interpersonal functioning in general.

## Introduction

Borderline personality disorder (BPD) is a serious and complex mental disorder characterized by pervasive difficulties with emotion regulation, impulse control, and instability in interpersonal relationships and self-image (Skodol et al., 2002). The lifetime prevalence of BPD is 5.9% (Grant et al., 2008). Since its earliest descriptions in the literature, impairment in interpersonal functioning has been a prominent characteristic of people with BPD, both from theoretical and diagnostic standpoints (Stern, 1938; Kernberg, 1967).

Despite the long history and that all current evidence-based treatments of BPD include strategies to improve interpersonal functioning, there remains a serious need to elucidate the specific interpersonal processes that are problematic for individuals diagnosed with BPD (Hill et al., 2011).

There are different methods that have been used for assessing interpersonal functioning in BPD individuals. Interpersonal functioning is traditionally measured by self-report questionnaires and interviews (Sinnaeve et al., 2015). Researchers have recently used other methods, such as experimental paradigms, behavioral observations, ecological momentary assessment, neuroscience based and psychophysiological tasks, with the aim to assess and characterize better interpersonal difficulties (see review Lazarus et al., 2014). However, most studies in BPD use unidirectional tests, such as concerning facial emotions expressed in pictures (Roepke et al., 2013; Lowyck et al., 2016). A disadvantage of both self-report questionnaires and current experimental paradigms is that the “hallmark of social interaction, the circular exchange of social signals between two or more individuals” (Roepke et al., 2013, p. 9) is not the focus of study. In this paper, we propose accompanied musical improvisation as an alternative method to investigate interpersonal processes associated with BPD. The embodied context of the musical interaction makes it possible to study the automatic, preconscious behavior within complex interpersonal interactions, which constitutes a lacuna in unidirectional tests.

Musical improvisation is frequently used in case studies to study interpersonal processes in music therapy with BPD patients (De Backer and Sutton, 2014). Clinical research in music therapy has a long tradition of qualitative research, based on detailed video and audio analyses of cases (Wheeler and Kenny, 2005; Lee and McFerran, 2015). The various methods and approaches that have been developed to study musical improvisations require many cycles of subjective listening and reflection in order to describe, analyze and interpret the therapeutic significance of the music (Bonde, 2005; Wosch and Wigram, 2007). Case study research from music therapy describes difficulties in musical interaction within the BPD population (Kupski, 2007; Knoche, 2009; Odell-Miller, 2011; Plitt, 2012; Hannibal, 2014; Strehlow and Lindner, 2016). Strehlow and Lindner (2016) described and categorized different interpersonal interaction dimensions of a music therapy process with BPD patients on the basis of an intensive case study (*n* = 20). Based on subjective analysis of music therapy video recordings, they identified 10 interaction patterns reflecting typical BPD themes such as splitting phenomena, trauma genesis, aggression and mentalization, and regulation of proximity and distance. One of the contributions of our study is to provide more objective, empirical evidence of the playing habits, and interpersonal behavior of BPD patients. For this, we will be using Music Information Retrieval (MIR) variables to quantify the playing habits and interpersonal behavior in musical improvisation with BPD individuals. To our knowledge, there is no existing research on the actual playing and interactions (i.e., interpersonal musical behaviors) of patients with BPD in music therapy.

### Attachment theory predicts impairments in temporal IPS in BPD individuals

Previous experimental research on musical improvisation has focused on individual performers (e.g., Keller et al., 2011; Norgaard, 2011, 2014). More recently, researchers have emphasized the interaction in joint improvisation as an ecologically valid domain to investigate interpersonal processes, and spontaneous coordinated behavior such as interpersonal synchronization (IPS) in particular (Keller et al., 2014; Walton et al., 2015). In a musical joint improvisation, the playing behavior emerges within a context of social collaboration, and without musical scores. Joint musical improvisation is a complex interaction to study, but Jeung and Herpertz (2014) stress the importance of socially complex stimuli to study interpersonal processes in patients with BPD.

Fundamental to the interactions involved in joint musical improvisation are affective and temporal IPS (Iyer, 2004; De Backer and Foubert, 2011; Hennig, 2014). Affective IPS in musical improvisations consists of shared moments that are important in changing the relationship and moving it to a deeper level of intersubjectivity within a therapeutic process. There have been a number of studies concerning affective IPS, addressing synchronicity (De Backer, 2008), meaningful moments (Amir, 1996), significant moments (Trondalen, 2006), affect attunement (Trondalen and Skårderud, 2007), and inter-affective synchronization (Schumacher and Calvet, 2007).

In this study, we will focus on temporal IPS. Temporal IPS entails the capacity to plan and execute specific actions at precise times, in relation to other performers. People can synchronize spontaneously, such as when people start to walk unintentionally in the same gait cadence. Other forms of temporal IPS can be intentional, for instance when dancers attune their movements to those of a partner. Temporal synchronization in a joint action is generally assessed based on measurements of “asynchronies” or timing deviations between people (Mills et al., 2015).

Experimental research in the normal healthy population demonstrates a strong relationship between the quality of temporal IPS in (musical) joint action and experiences related to social cohesion (Marsh et al., 2009), cooperation (Anshel and Kipper, 1988; Wiltermuth and Heath, 2009), bonding and attachment (Hove and Risen, 2009; Wheatley et al., 2012). As for the BPD population, individuals appear to cooperate less in an experimentally manipulated interpersonal context than do controls (Lazarus et al., 2014). Further, BPD individuals are likely to have more difficulties in repair of relationship ruptures than controls (King-Casas et al., 2008). Ruptures in cooperation seem to be associated with diminished trust in the interacting partner (Seres et al., 2009; Unoka et al., 2009). Finally, oxytocin, a neuropeptide known to enhance cooperation and prosocial behavior for instance in musical joint action (e.g., Grape et al., 2002), may have paradoxical effects for BPD individuals. For example, a study of Bartz et al. (2010) showed that intranasal administration of oxytocin did not have its normal trust facilitating effects in response to a hypothetical partner cooperation in BPD individuals.

From a theoretical viewpoint, BPD is typically characterized by disturbed attachment (Agrawal et al., 2004; Gunderson and Lyons-Ruth, 2008; Beeney et al., 2016). According to attachment theory (Bowlby, 1988), the quality of relationships, such as measured by child-caregiver IPS, results in the development of mental representations, including beliefs about the self, expectations about interpersonal relationships and their quality, all of which act as prototypes or attachment patterns (e.g., secure/insecure) in later adult social interactions (Fraley, 2002; Shaver and Mikulincer, 2005; Scott et al., 2009; Lindsey and Caldera, 2014). This attachment theory is supported by research suggesting that the quality of child-caregiver IPS is critical to the emergence of other socio-cognitive and socio-affective abilities (Crandell et al., 2003; Feldman, 2007a; Newman et al., 2007; Feldman, 2007b, 2012; Gratier, 2009; Hobson et al., 2009; Guedeney et al., 2011; Kiel et al., 2011; Kleinspehn-Ammerlahn et al., 2011; Dumas et al., 2014).

Empirical research shows that BPD patients have difficulties in maintaining close relationships, and attachment relationships in particular (e.g., romantic partner, Melges and Swartz, 1989; Levy, 2005; Gunderson and Lyons-Ruth, 2008; Choi-Kain et al., 2009; Fonagy and Luyten, 2009; Beckes and Coan, 2011; Levy et al., 2015; Beeney et al., 2016). Difficulties in attachment relationships are characterized by oscillations between opposing fears of abandonment and dependency, between neediness and angry withdrawal (Melges and Swartz, 1989). This leads to unstable relationships and difficulties in maintaining strong attachments with others (Bodner et al., 2011). For example, a recent study by Lazarus and Cheavens (2016) found that women with BPD reported more relationship ruptures within the previous month compared to healthy control women.

Based on attachment theory and associated empirical research, we predict that in our study involving an accompanied musical improvisation, the (insecure) attachment system will be activated in BPD patients, and this will affect temporal IPS between therapist and BPD patients. More specifically, we predict:

1. poorer temporal IPS, represented by higher overall timing deviations, for BPD patients compared to normal controls;
2. more oscillations (e.g., more variability) in timing deviations between therapist and BPD patients compared to normal controls;
3. problems in maintaining and improving IPS between therapist and BPD individuals in the course of the joint improvisation compared to normal controls.

### Impulsivity traits predict differences in temporal IPS in BPD individuals

Additionally, from the perspective of BPD pathology, we assume that impulsivity, a core feature of BPD, will influence temporal IPS in a joint musical improvisation. Impulsivity is one of the 9 diagnostic criteria in the Diagnostic and Statistical Manual of Mental Disorders, 4th edition (DSM-IV; American Psychiatric Association, 1994). In the literature, BPD is often described and conceptualized as a disorder characterized by high levels of impulsivity (Silk, 2000; Depue and Lenzenweger, 2001; Widiger and Costa, 2002; Scott et al., 2009). These findings motivate a further prediction for our own study, that (4) BPD patients will play in a more impulsive manner than normal controls. In other words, we predict that BPD individuals will be less inhibited in their playing than normal controls and adapting to the therapist's playing more readily.

## The present study

In this study, we propose using MIR variables for investigating how aspects of a participant's piano playing vary across an accompanied improvisation. Generally, the field of MIR is concerned with the extraction of meaningful information from musical content (Peeters, 2013). Relevant existing work includes research on performance style analysis (Dannenberg et al., 1997; Widmer, 2002; Widmer and Goebl, 2004; Stamatatos and Widmer, 2005; Cheng and Chew, 2008; Chew, 2012), temporal coordination between performers (e.g., Loehr and Palmer, 2011; Keller et al., 2014; Washburn et al., 2014), and one improvisation study involving people with mental retardation (Luck et al., 2006). Luck et al. (2006) found significant associations between musical behavior and diagnosis level—that “most of the features that predicted level of mental retardation related to temporal aspects of the clients' improvisations” (p. 43). We use MIR variables to measure the presence and development of temporal IPS between accompanist and participant, and to measure the presence of rhythmic motifs/patterns in participants' playing. The temporal IPS variables overlap with those used in previous work (e.g., Widmer and Goebl, 2004; Luck et al., 2006; Loehr and Palmer, 2011), although, as we are motivated by different aspects of theory and are therefore investigating different predictions, there is not necessarily a one-to-one correspondence between variable definitions. It is the application of these variables in the context of BPD and joint improvisation that is novel.

Work on performance style analysis and temporal coordination between performers tends to identify temporal, dynamic, and articulatory variable categories. There is a focus on how performers vary in playing the same piece, with less attention paid to *what* notes are played, since this is the same or very similar across performances. Relative to this literature, the variables we calculate include some novel quantifications of *what* is being played—of rhythmic motifs/patterns, based on previous investigations into automatic pattern discovery in music (Collins et al., 2010, 2016; Collins and Meredith, 2013). While our hypotheses are concerned mainly with temporal IPS, it could be that aspects of attachment style and impulsivity manifest not so much in timing information as in other dimensions of musical organization.

Taking the introduction and this section on MIR variables together, in this study we propose a novel structured piano improvisation paradigm and MIR variables to investigate how aspects of temporal IPS vary across the improvisation. We use logistic regression modeling with these MIR variables as independent variables, to predict whether a given participant is a patient or a control, as well as to address our predictions (1)–(4).

## Methods

### Participants

A sample of 16 carefully screened BPD patients and 12 matched normal controls participated in the study. Participants in the BPD group were patients consecutively admitted in the psychiatric hospital UPC KULeuven, Kortenberg (Belgium), who met the following inclusion criteria: (a) a primary diagnosis of BPD according to the structured clinical interview for Diagnostic and Statistical Manual of Mental Disorders, fourth edition (DSM–IV) Axis II disorders (SCID-II), (b) age between 21 and 60 years, and (c) not participated in music therapy sessions previously. Twenty eight patients were screened for BPD, 20 patients who fulfilled the inclusion criteria were asked to participate in the study, and, subsequently, 16 patients confirmed willingness to do so.

Participants in the matching normal control sample were recruited from the community, based on characteristics from the BPD group. Control participants were pairwise-matched with the BPD sample on gender, age, level of education, level of musical education, and musical principal instrument. Sixteen potential participants were asked and agreed to participate. Four of these participants were excluded because they fulfilled criteria for at least one personality disorder based on the Assessment of DSM-IV personality Disorders (ADP-IV), a self-report questionnaire for personality pathology (see below).

In summary, we collected and carried forward to the analysis data from 16 BPD patients and12 matched controls. The absence of matches for four BPD patients was not of particular concern, because from a methodological standpoint, even if matching has been performed, this does not necessitate a matched analysis (Pearce, 2016).

The study was carried out in accordance with the recommendations of the local ethics committee, UPC KULeuven, and the central ethics committee, UZ KULeuven. All subjects gave written informed consent in accordance with the Declaration of Helsinki. After being provided with the necessary information, all participants (BPD group and matched normal control group) signed informed consent forms and were given an appointment to participate in the improvisation within 4 days. After completing the musical improvisation, participants were asked to fill out the questionnaires as detailed below.

Most of the BPD patients were female (12 female; 3 male; 1 transgender). The mean age was 31 years (sd = 9.41; range 21–51). Three patients completed primary high school, four secondary high school, six higher education (professional bachelor), and three higher education (academic master). Seven patients indicated experience of playing a musical instrument, among which five patients indicated that they had received musical education and two patients described themselves as autodidacts. Two patients had 1 year of musical education; two patients had 2 years of musical education; one patient had 7 years of musical education; one patient had one and a half years autodidactic experience; and one patient had 7 years autodidactic experience. Three patients indicated voice as principal musical instrument, two patients played guitar, one patient piano, and one patient flute.

At the time of the study, 50% of the BPD patients were receiving psychotropic medication. Most patients were using more than one type of psychotropic medication (*n* = 5); only three patients (19%) were using one type of psychotropic medication.

### Questionnaires and measurements

Both BPD patients and normal controls completed (a) a listening test of beat perception, (b) a questionnaire assessing age, gender, educational level, musical principal instrument, musical educational level, music therapy history, motoric restrictions, hearing problems, and sensitivity for sound, (c) self report measures of musical sophistication to analyse the confounding influence of musical experiences (d) self report measures of attachment (see below). Data on depression and current psychotropic medication were only gathered for the BPD group. Our reasoning for testing the influence of depression was because depression has been hypothesized as a possible confounder in interpersonal functioning in BPD (Fonagy and Bateman, 2008; Lowyck et al., 2016). Current psychotropic medication was gathered based on the medical records of the BPD patients.

#### The goldsmiths musical sophistication index (Gold-MSI)

The Goldsmiths Musical Sophistication Index (Müllensiefen et al., 2014) is a self-report inventory for individual differences in musical sophistication. Because no Dutch translation was available, we made use of a back-translated design (Hambleton, 2005) to provide a Dutch translated version of the test. Gold-MSI is a 38-item self-report questionnaire. A range of musical skills, abilities, and behaviors are measured which are observable in both musicians and non-musicians. The Gold-MSI assesses General Musical Sophistication and includes additional five subscales: Active Engagement, Perceptual Abilities, Musical Training, Singing Abilities, and Emotions.

#### Beat alignment test

The Iversen and Patel's (2008) beat alignment test is a beat perception test that includes 18 short fragments of instrumental music (each excerpt 10–16 s in duration). The 18 excerpts originate from nine musical pieces within three different styles: Rock, jazz, and well-known classical. The tempi of the short excerpts have a range 85–165 BPM. Participants were invited to listen to the excerpts and to respond whether a simultaneous beep track was on or off the beat of the music. Half of the excerpts had beep tracks exactly on the beat of the music, the other excerpts had beep tracks off the beat.

#### Diagnostic inventory for depression (DID)

The DID (Zimmerman et al., 2004) is a 38-item self-report scale. Both severity of depression and symptom frequency are assessed based on DSM-IV criteria. From this study, we used only the nineteen-item severity subscale. The DID has high levels of test-retest reliability, and good convergent and discriminant validity. The DID was only administered in the BPD group.

#### Structured clinical interview for DSM-IV Axis II disorders (SCID II)

The SCID II interview (First et al., 1997), in a dutch translated version (Weertman et al., 2000), consists of 119 questions assessing the DSM-IV personality disorders (i.e., paranoid, borderline, narcissistic, schizoid, schizotypal, antisocial, histrionic, avoidant, dependent, and obsessive compulsive). We administered a selection of the SCID, namely the questions assessing borderline personality disorder (15 questions). The SCID-II was only registered in the BPD group and was executed by a senior psychologist, trained in the assessment of the SCID-II interview.

#### Assessment of DSM-IV personality disorders (ADP-IV)

The ADP-IV (Schotte et al., 1998) was administered in the BPD group and the control group as a screening tool to detect potential personality pathology. The ADP-IV is a screening tool for personality disorder and includes 94 items in a randomized order, which represent 80 criteria of the 10 DSM-IV personality disorders, as well as two personality disorders listed in the DSM–IV for research purposes (the depressive and passive-aggressive personality disorders), which are represented in additional 14 research criteria. Each item is rated on a seven-point trait scale, from 1 (*totally disagree*) to 7 (*totally agree*). When a person recognizes the presence of a trait and is giving a score of five (*rather agree*) or higher on a trait question, he/she is asked to answer an additional distress question, “Has this characteristic ever caused you or others distress or problems?” His/her additional answer is scored on a three-point scale: 1 (*totally not*), 2 (*somewhat*), 3 (*most certainly*). The ADP-IV provides dimensional and categorical scoring formats. Categorical personality disorder diagnoses are acquired according to the DSM-IV thresholds. In this study we used the categorical scoring format. Control subjects were excluded in this study when they scored above the respective DSM-IV thresholds.

#### Relationship structures (ECR-R)

The Relationship Structures questionnaire (Fraley et al., 2011) is a self-report measurement that is designed to assess two fundamental dimensions underlying attachment patterns: Anxiety and avoidance (Fraley et al., 2000). The anxiety dimension assesses the extent to which people have the tendency to worry about attachment-related concerns, such as the availability and responsiveness of an attachment figure. The avoidance dimension assesses the extent to which people have the tendency to depend on others and to be uncomfortable opening up to them. Prototypically secure people tend to score low on both anxiety and avoidance dimensions. BPD patients tend to score high on anxiety dimensions (Levy, 2005; Levy et al., 2015). The measurement has 9 items and is developed with the aim to assess patterns of attachment across several distinct relationships (mother, father, romantic partner, and best friend). Participants were asked to indicate for each item on a seven-point scale the extent to which they agreed or disagreed with the statement (1: *strongly disagree*; 7: *strongly agree*). The same 9 items can be used with the distinct relationships described above. Recently, a new supplementing item set was designed to assess people's general attachment styles (Fraley et al., 2015). The 9 items can be used also to assess only one kind of relationship, which is described as a short 9-item version of the ECR-R. We included one set of 9 items to assess only one relationship style: People's general attachment styles. This was administered both in the BPD group and the normal control group.

### Stimuli

**Figure 1:**
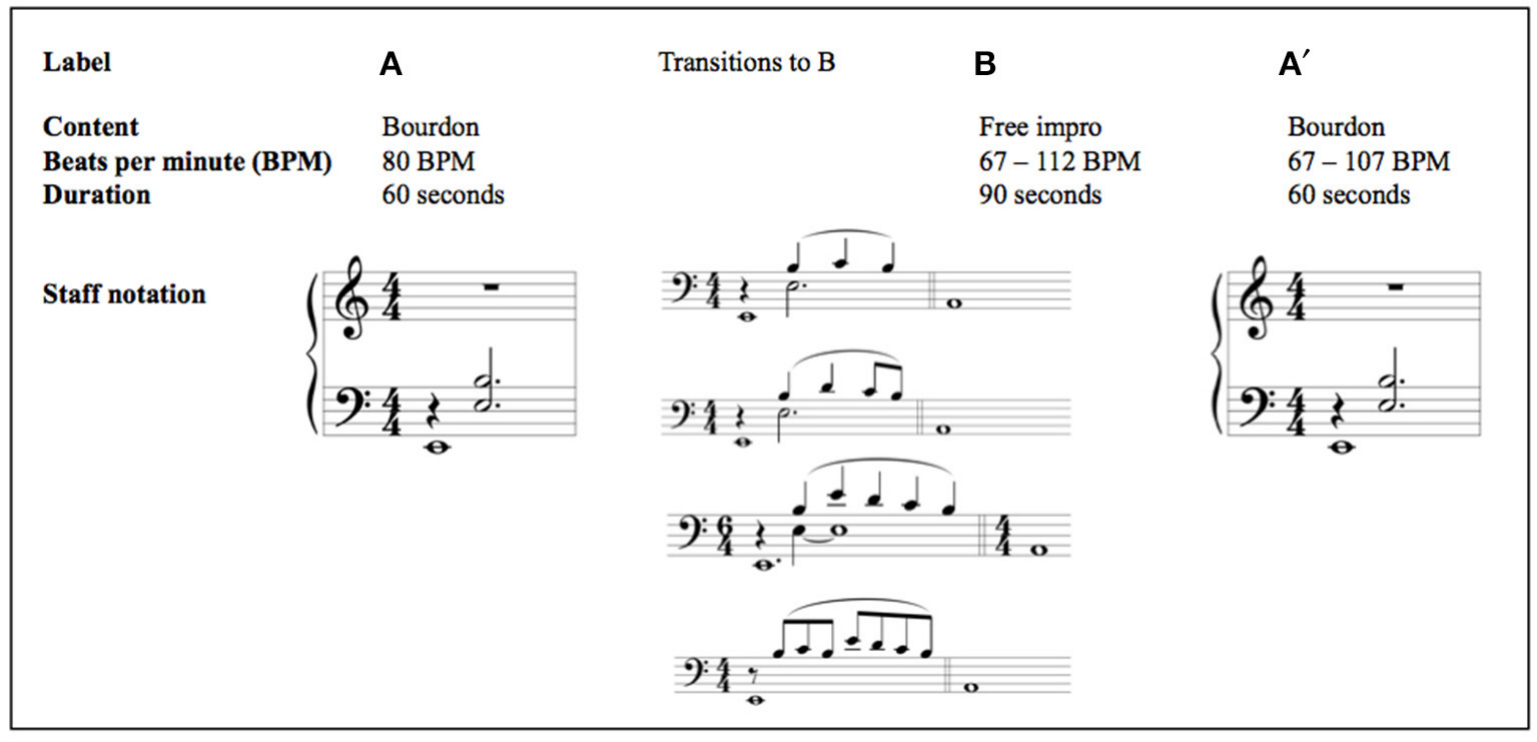
**This figure represents the ABA′ structure of the accompaniment design, with characteristics in content, beats per minute (BPM), and duration**. To make the transition to part B, the improviser added a short melodic phrase above the bourdon, which initiates the new character and mode. The staff notation excerpts contain the bourdon accompaniment figure, as well as the most frequently played transitions from A to B in this study.

We use a novel, structured piano improvisation paradigm distinguishing between two different accompaniment frameworks—a predictable repetitive interaction, and a more dynamic, socially complex interaction. The therapist's accompaniment was designed to be in a three-part ABA′ structure (see Figure 1): In part A, the accompanist played a single low note that sounded for one beat before a two-note chord was played and both were sustained for three beats to make up a four-beat pattern (see the staff notation below A in Figure 1). The musical term for this type of accompaniment figure is “bourdon,” and it was repeated throughout section A at a steady tempo; as implied by the label A', this bourdon pattern returned in the third section of the therapist's accompaniment; the content of section B was somewhat freer, but it generally contained an increase in tempo and dynamic level, as well as a change from Phrygian to Aeolian modes.

The rationale for this accompaniment design is that in the more dynamic B part of the improvisation, the interaction comes to the fore. Our premise is that in part B, the attachment system will be more activated in BPD patients than in either parts A or A'. As such, differences in temporal IPS between patients and controls may well be revealed in the B part of the accompanied improvisation. The most convenient way to determine whether changes in IPS have occurred within part B is to split the music data for this section in two, B1 and B2, and calculate variables for these subparts separately. In experiments on visual working memory (e.g., Brady et al., 2009), it is quite common to establish regularities in stimuli, upon which participants may come to rely in order to improve task performance, before subverting those regularities and measuring participants' sensitivities. Our ABA′ accompaniment structure, where A establishes the regular bourdon and B subverts it, can be seen as a less common and therefore relatively novel musical analog of experimental paradigms that establish and then subvert regularities in order to measure participants' sensitivities.

As mentioned above, we made use of an ABA′ structured piano improvisation, distinguishing between two different accompaniment frameworks. In the next section, we will give more music-theoretic details related to our improvisation design.

#### Part A

The A part of the piano improvisation is defined by a repetitive bourdon figure (as presented in Figure 1). A bourdon is a sustained or repetitive tonic tone of a scale or mode. When a tone a fifth above the tonic is added (as in Figure 1), one speaks of a “fifth bourdon.” The technique of sustaining a tone or fifth is originally derived from folk music, wherein melodies were developed over a sustained fifth bourdon. The advantages of using a fifth bourdon are as follows: (1) it provides a technically simple accompaniment with a harmonic basis; (2) this basis is flexible with respect to mode (e.g., an elaboration might be major/minor, modal, or atonal); (3) bourdon offers many possibilities for the development of a melody or a polyphonic elaboration by a participant.

**Figure 2:**
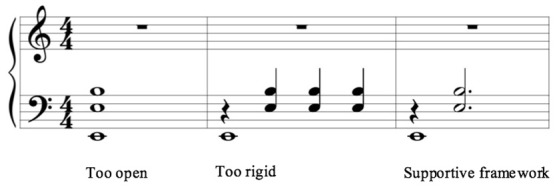
**Different options of rhythmic articulation for the bourdon accompaniment**.

The bourdon can also hinder musical elaboration/improvisation when its use is too open (Figure 2, left) or too rigid (Figure 2, center). Therefore, the bourdon in this context is articulated with a metric pulse on the first and second beat in common time (Figure 2, right). When a meter manifests itself as such, the participant may experience this as a supportive framework for improvisation.

Participants were instructed to play only the white keys of the piano. This constrained the tonal scope of the improvisations somewhat, but still left open the possibility that the participant might emphasize (implicitly or explicitly) one or more modes (e.g., Ionian by emphasizing pitch-class C, Dorian by emphasizing pitch-class D, etc.). Taken as a whole with the therapist's bourdon (which emphasizes pitch-class E), the implied mode may well be E Phrygian. The Phrygian mode, while having a distinctive sound, can be found in a lot of musical cultures (in Japanese scales, Spanish music, jazz, etc.).

Finally, we chose a playing speed of 80 BPM. This was indicated to the therapist via a beep sound (rather, than say, a blinking light), just before the improvisation began. This was done aloud to indicate the speed to the participant also. As measured in adults, this tempo is more toward the lower boundary of speeds to synchronize with an external pulse (Drake et al., 2000). We chose this lower speed because several of the participants had little piano playing or musical experience, so the slow speed gave them the opportunity to explore the instrument without the pressure of a faster tempo. We expected an accelerando (speeding up) in the B part of the improvisation.

#### Transition and part B

Part B of the improvisation always starts in A Aeolian, following a brief transition. There was only one exception to this in all our data. This new and clear mode constitutes a substantial change after the repetitive A section with its open fifths character. To make the transition to the B part, the improviser adds a short melodic phrase, above the fifth bourdon, which initiates the new character part B (see Figure 1). The B part is characterized by relatively little repetition and freer improvisation. The tonal content remains modal, however. In this section, the therapist was asked to attune and adapt his playing (tempo, timbre, and dynamic) to that of the participant. Generally, we observed an initial increase in tempo and dynamics in this section.

#### Part A′

The A′ part of the improvisation was a return to the repetitive bourdon figure of part A, the only difference being that generally the tempo began higher (due to coming from the faster B part), and we placed no restriction on it returning to 80 BPM (although sometimes it did).

### MIR variables

In the previous section, we stated the potential utility of MIR variables for investigating how aspects of a participant's piano playing vary across an accompanied improvisation. In the interests of clarity, we defer details of the music data processing and mathematical definitions of all MIR variables to Supplementary Materials. In brief, the music data was beat-tracked by a professional musician/music therapist, and each improvisation was then quantized automatically using the Lisp package MCStylistic and Matlab package PattDisc (Collins, 2011). The purpose of these steps (beat-tracking and quantization) is to map and/or compare each performed note to a start time (called ontime) commensurate with how it would be written in staff notation, as a basis for measuring participants' IPS. The variables we considered are shown in Table 1 (see Supplementary Materials for full definitions). Below we mention only those that became most relevant in our analyses. To avoid our analyses becoming too exploratory, we employed a common, principled variable selection technique called stepwise selection. As can be seen from Table 1, the focus was on variables associated with IPS (seven out of 15), but for the sake of thoroughness we included several from other categories (tempo, rhythmic patterns, and interpersonal imitation) that were either straightforward (Occam's razor) or could be obliquely related to IPS.

**Table 1:** **Summary of the MIR variables used to quantify aspects of a participant's playing in the accompanied improvisation**.

**No**.	**Variable label**	**Variable name/Description**	**Relation to theory**
1	MD_m	Mean metrical deviation	IPS
2	MD_sd	Standard deviation of metrical deviation	IPS
3	LP	Lag proportion	IPS
4	MDA_m	Mean of metrical deviations that are ahead of the beat	IPS
5	MDA_sd	Standard deviation of metrical deviations that are ahead of the beat	IPS
6	MDB_m	Mean of metrical deviations that are behind the beat	IPS
7	MDB_sd	Standard deviation of metrical deviations that are behind the beat	IPS
8	TMP_m	Mean tempo	Tempo
9	TMP_sd	Standard deviation of tempo	Tempo
10	CR_dur	Compression ratio applied to (ontime, duration)-pairs	Rhythmic patterns
11	TC_o	Translational coefficient applied to ontimes	Rhythmic patterns
12	RS	Rhythmic simplicity	Rhythmic patterns
13	DN	Note density	Interpersonal imitation
14	AI_mu	Mean articulation interaction	Interpersonal imitation
15	AI_min	Minimum articulation interaction	Interpersonal imitation

*The final column indicates the aspect of theory to which a particular variable may be most relevant*.

**Figure 3:**
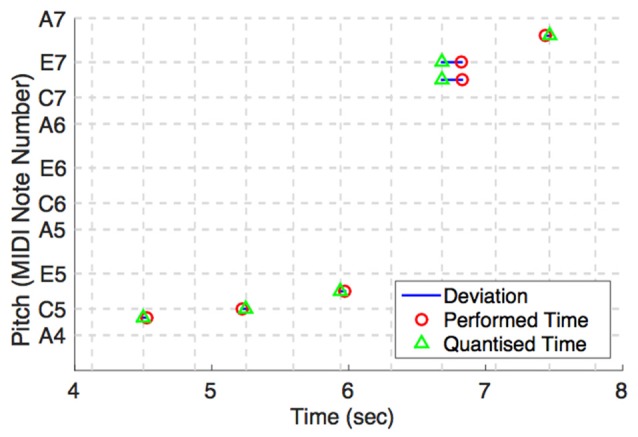
**Metrical deviations as a behavioral measure of IPS**. Horizontal dashed lines indicate the MIDI note numbers of important pitches in A Aeolian. Vertical dashed lines indicate eighth-note beats. On the eighth-note beat before 7 s, there is a relatively large timing deviation between the participant's and therapist's playing. It can be seen that the participant's performed time of two notes (red circles) lags behind that of the therapist's (green triangles). The blue lines are a visual aid to indicate the deviation from the closest eighth-note beat.

(1) MD_m stands for mean metrical deviation. This calculates the average deviation between each note performed by a participant and the underlying eighth-note beat of the therapist to which it is closest. It is indicated by the blue horizontal lines in Figure 3. The larger the value of MD_m, the more the participant deviates from the beat over an improvisation section, and the more “out of time” their playing will sound. We use this as one operational definition for the participant's overall asynchronies.

(2) The above variable says nothing about whether the participant tends to play ahead of or behind the beat, or leader-follower behavior. The variable LP, standing for lag proportion, is the proportion of times that a participant's notes are behind the beat over the course of an improvisation section. If a participant is always ahead of the beat, then LP = 0, and thus the participant shows more leader behavior; if a participant is always behind the beat, then LP = 1, and thus the participant shows more follower behavior.

(7) The variable MDB_sd, standing for standard deviation of metrical deviations behind the beat, measures the consistency of timing deviations of those notes played late by the participant. If a participant tends to play late (behind the beat) in a consistent manner, then this variable will take a relatively small value; if a participant tends to play late in an erratic manner, then this variable will take a relatively large value.

(10) CR_dur stands for compression ratio applied to (ontime, duration)-pairs. Existing work posits that the more it is possible to compress data, the more structure or patterning the original data contains (Collins et al., 2010, 2016; Collins and Meredith, 2013). The more rhythmic motifs or patterns in a participant's playing, the more their corresponding (ontime, duration)-point set tends to be compressible, and the higher the compression ratio will be.

(12) The variable RS, standing for rhythmic simplicity, measures the prevalence of a participant's most prevalent rhythm. We tally their inter-onset times (the time differences between the notes played), determine their modal (most prevalent) time difference, and define RS as the proportion of all time differences that belong to this mode. If a participant plays only isochronous (evenly spaced) notes (possibly of differing pitches), then their RS = 1; if a participant plays *n* notes such that the time difference between two consecutive notes is never the same, then RS = 1/*n*, i.e., is close to 0.

Variables were calculated from the separate parts of the accompanist's ABA′ structure, with the additional bisection of section B into B1 and B2 (on the basis of the overall duration of part B), to enable investigation of participant sensitivities to the changes in musical content at the beginning of section B.

### Apparatus

The piano improvisation was recorded using a Yamaha Disklavier MPX70 piano. Each key was connected with a specially designed optical sensor, and these were connected to a USB MIDI interface (Motu Midi Express 128). Improvisations were recorded with Logic Pro X (Mac system) and exported as MIDI files for subsequent analyses. The MIR variables were calculated in Matlab, and R was used for conducting statistical analyses.

### Procedure

Participants were asked to play intuitively and freely on the piano's white keys, without playing well-known songs, but with the aim of exploring joint interaction with the accompanist. They were informed about the ABA′ structure of the improvisation.

The accompanist was a senior registered music therapist, and undertook all the improvisations. The accompanist was blind in the sense that no knowledge about the background of the participants (control or BPD group) was known. The accompanist had 35 years of clinical experience, was experienced in the use of clinical improvisation, and had expertise in the therapeutic musical interventions described by De Backer et al. (2014).

The piano was chosen as an instrument based on a previous study about choices of musical instruments used in individual music therapy sessions with BPD patients (De Backer et al., 2016). In that study, seven Belgian music therapists were asked to fill in questionnaires about the musical instruments chosen by BPD patients in individual sessions over a period of 1 year. Piano was the most frequently used instrument in this population.

**Figure 4:**
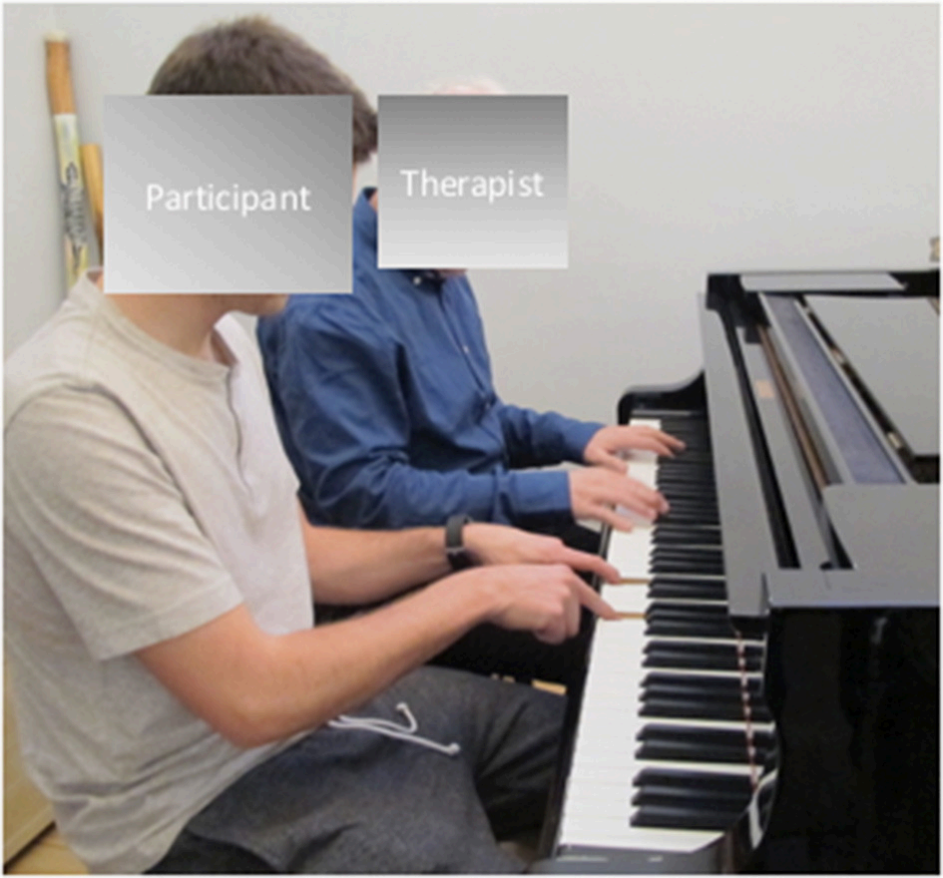
**Piano improvisation setting**. Participant and therapist play together on one piano. The figure shows the position of the therapist, toward the left side of the keyboard, and the participant, toward the right side of the keyboard (when looking from behind them).

The accompanist was sitting on the left side of the piano and played the lower registers of the keyboard. The participant was sitting on the right side, and was playing the upper registers (as shown in Figure 4). This setting was based on the concept of the left and right hand position within music therapy (De Backer et al., 2014)—that the therapist can sustain and support (harmonically) the play of the participant. The keyboard had a split point on G4, which enabled (mostly) convenient splitting of the therapist and participant's playing in Logic.

## Results

**Figure 5:**
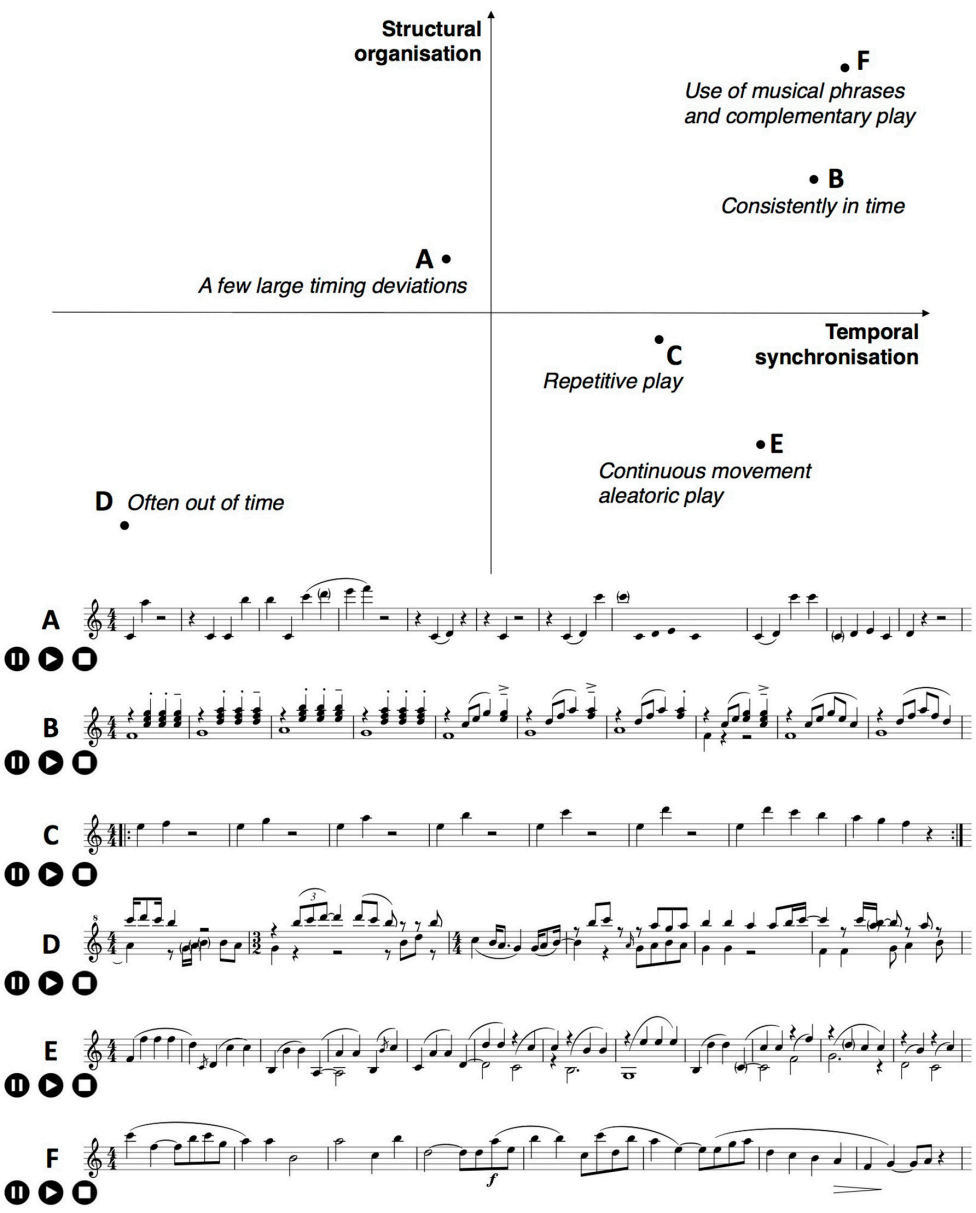
**A plot to describe musical characteristics of excerpts from recorded musical improvisations, with structural organization of musical notes on the vertical axis and temporal synchronization on the horizontal**. Excerpts **(A–F)** below the plot are transcriptions of the excerpts into staff notation. A clickable version of this plot is available in the online version of this paper.

We conducted both musical and statistical analyses of our data^1^. In terms of musical analysis, Figure 5 (clickable in the online version of the paper) shows transcriptions of some representative excerpts and a plot of how they might be located in a two-dimensional space consisting of temporal synchronization and structural organization. There is clear evidence of distinct playing habits, but these transcriptions and plot were made primarily to deepen our knowledge of participants' improvisations, rather than to address any of our four predictions directly.

### Statistical analysis with MIR variables

In terms of statistical analyses, we conducted logistic regressions on a dependent variable of BPD (patient = 1, control = 0), using independent variables as described in the section “MIR variables.” In other words, we investigated whether it was possible to predict the category of a given participant (BPD or control), based solely on quantifications of their playing habits. Initially, we employed a stepwise selection procedure. Incorporated in the first stage of stepwise selection is a comparative assessment of the discriminative power of each variable in isolation. Supplementary Figure 2 shows the distribution for each variable, split into patients and controls, and that there was only one variable (lag proportion in section B1, or LP_B1) with significant differences between BPD_patients and controls, [*t*_(25.04)_ = −2.32, *p* = 0.029]. As such, LP_B1 is the first variable to enter the model.

While rigorous, stepwise selection and the inclusion of further variables tended to result in overfitting of the data and coefficient blowup.^2^ En route to overfitting, however, we identified a parsimonious model that provided strong predictions of BPD or control, as summarized in the following equation and Table 2A. The model consists of mean metrical deviation in section B1 (MD_m_B1) and mean metrical deviation in B2 (MD_m_B2):

$$\begin{array}{l} y=3.35-257.44\text{ MD}\_\text{m}\_\text{B}1+210.28\text{ MD}\_\text{m}\_\text{B}2+\varepsilon \end{array}$$

where *y* is the log odds of having BPD (patient ≈ 1, control ≈ 0) and ε is an error term. Nagelkerke's *R*^2^ = 0.57 for this model, and the Hosmer-Lemeshow test indicates that the actual diagnoses (patient or control) are not significantly different from those predicted by the model, χ^2^(8) = 5.31, *p* = 0.72. The signs of the coefficients, −257.44 and +210.28, are opposite, suggesting it is the difference between metrical deviation in sections B1 and B2 that drives prediction of borderline personality disorder. On further inspection, patients' metrical deviations either tended to become bigger in section B2 than in B1, or remain the same, meaning their log odds of having BPD was driven toward 1 in the above formula by the constant term being not much reduced by −257.44 × MD_m_B1 + 210.28 × MD_m_B2. Controls, on the other hand, had smaller metrical deviation in B2 than in B1, meaning their log odds of having BPD was driven toward 0 by −257.44 × MD_m_B1 being negative and of greater magnitude than 210.28 × MD_m_B2.

**Table 2:** **Summary of three binary regressions on (A) mean metrical deviation in section B1 (MD_m_B1) and the same in section B2 (MD_m_B2), (B) lag proportion in section B1 (LP_B1), and (C) lag proportion in section B1 (LP_B1) and mean metrical deviation in section B2 (MD_m_B2)**.

**Variable**	***B***	***SE B***	***z*-value**	***P***
**A**				
Intercept	3.35	2.10	1.60	0.111
MD_m_B1	−257.44	103.40	−2.49	0.013
MD_m_B2	210.28	95.46	2.20	0.028
Null deviance: 38.24 on 27 degrees of freedom				
Residual deviance: 22.80 on 25 degrees of freedom				
AIC: 28.80				
**B**				
Intercept	3.55	1.71	2.08	0.038
LP_B1	−7.66	3.83	−2.00	0.045
Null deviance: 38.24 on 27 degrees of freedom				
Residual deviance: 33.16 on 26 degrees of freedom				
AIC: 37.16				
**C**				
Intercept	5.73	2.47	2.32	0.021
LP_B1	−8.81	4.39	−2.01	0.045
MDB_sd_B1	−37.86	24.06	−1.57	0.116
Null deviance: 38.24 on 27 degrees of freedom				
Residual deviance: 30.13 on 25 degrees of freedom				
AIC: 36.77				

*The second column B, contains the coefficient estimate, the third column SE B, contains the standard error of that coefficient, the fourth column contains the z-value and the fifth column the associated p-value. As well as reporting null and residual deviances for each model, Aikaike's information criterion (AIC) is reported also. Models with lower AIC are said to have a better fit to the data, while taking into account the number of constituent variables*.

**Figure 6:**
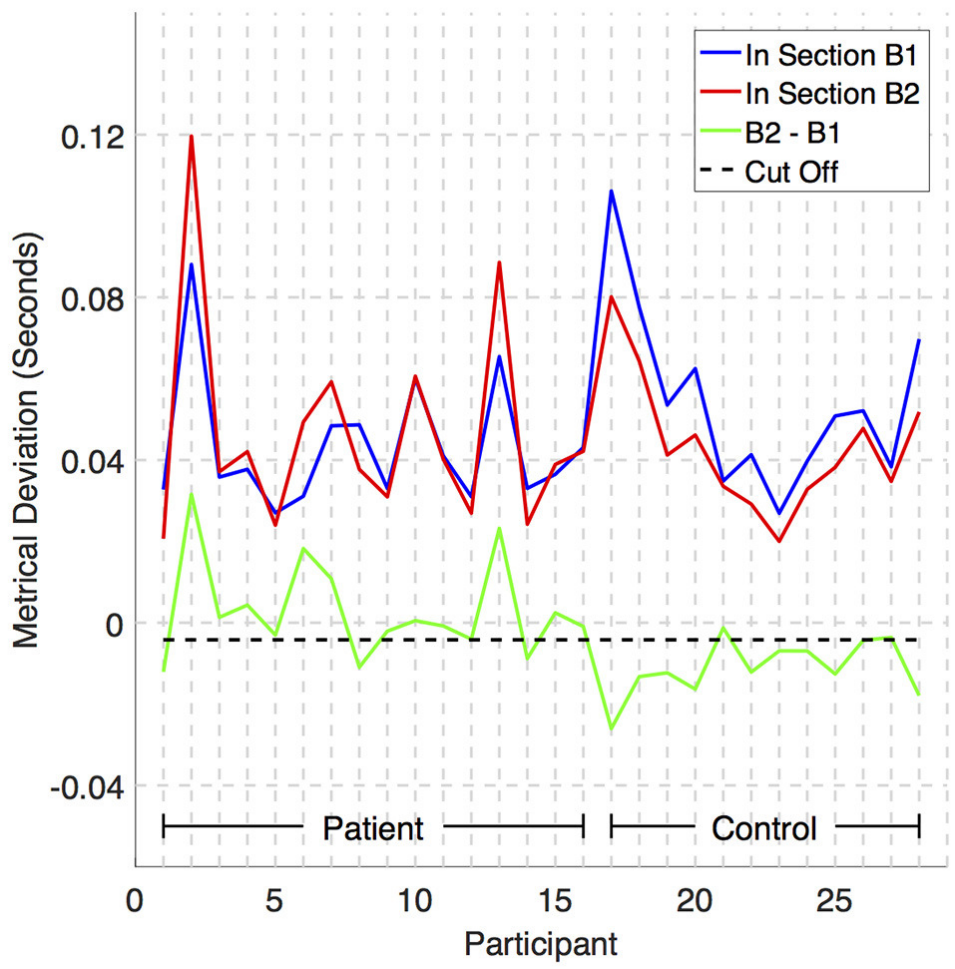
**A plot of mean metrical deviations against participant**. Mean metrical deviation MD_m is the mean of the absolute deviations between each played note onset and the time of the closest underlying beat. The blue line is MD_m for section B1, the red line is MD_m for section B2, the green line is the difference MD_m_B2−MD_m_B1, and the black horizontal line indicates a cut off that discriminates between most BPD patients and controls.

A plot of metrical deviation in section B1 is shown in blue in Figure 6, metrical deviation in section B2 is shown in red, the difference MD_m_B2−MD_m_B1 is shown in green, and a dashed black line indicates how the difference acts as an effective discriminator between BPD patient and control. All but three patients have a difference above the cut off and all but two controls have a difference below. While the difference may seem small (e.g., cut off is ~4 ms), the pattern in results in Figure 6 is clear: The blue line is always above the red for controls, but not always so for BPD patients.

Applying a leave-one-out cross-validation procedure with this model, we found a prediction error of 0.18. In other words, this model successfully predicts whether a given participant has or does not have BPD in 23 (= 0.18 × 28) out of 28 cases (82% success). The chance that a baseline (guessing) model succeeds in predicting more than 18 cases is less than 0.05 [*P*(*B*_*n* = 28, *p* = 0.5_ > 18) = 0.043]. Even if we “assist” the baseline model further, by including the knowledge that the proportion *p* of BPD patients is 16/28, success in predicting more than 20 cases is less than 0.05 [*P*(*B*_*n* = 28, *p* = 16/28_ > 20) = 0.040]. That is, our predictive model for BPD performs significantly better than chance.

As suggested by the first stage of stepwise selection, another model that might provide strong predictions of BPD is based on lag proportion in section B1, LP_B1 (Table 2B). Nagelkerke's *R*^2^ = 0.22 for this model, and the Hosmer-Lemeshow test indicates that the actual diagnoses are not significantly different from those predicted by the model, χ^2^(8) = 4.77, *p* = 0.78. The negative coefficient on LP_B1 suggests that the more a participant lags behind the beat in section B1, the more likely that participant is to be a control. AIC was not as good for the lag variable (= 37.16) as for the model in Table 2A (AIC = 28.80), however, and also prediction error on cross-validation was worse (0.24). To investigate whether we might improve the lag proportion model further, we built a third model based on it and variation in playing behind the beat in section B1 (MDB_sd_B1, see Table 2C). Nagelkerke's *R*^2^ = 0.34 for this model, and the Hosmer-Lemeshow test indicates that the actual diagnoses are not significantly different from those predicted by the model, χ^2^(8) = 4.45, *p* = 0.81. This was motivated by seeding a stepwise selection procedure with LP_B1 and including the strongest predicting variable in the next stage, which happened to be MDB_sd_B1. Despite the inclusion of an extra variable, AIC (= 36.77) was not as low as for the metrical deviation model in Table 2A (AIC = 28.80). The MDB_sd_B1 variable was not significant in its own right (*p* = 0.116 in Table 2C) and prediction error on cross-validation was worse (0.23).

Overall, therefore, we recommend the metrical deviation model as a parsimonious and, according to cross-validation, robust predictor for BPD. As described above, we explored various possibilities in an attempt to find a better model. Now we use the *t*-test results mentioned briefly at the beginning of this subsection to address questions of significant differences between patients and controls for the metrical deviation (MD_m_B1) and lag (LP_B1) variables: (1) is there a significant difference in MD_m_B1 between BPD patients and controls? According to Welch's two-sample *t*-test, there is no significant difference [*t*_(19.28)_ = −1.49, *p* = 0.153]. If we restrict the data to matched participants so that we can conduct a (generally more powerful) paired *t*-test, still there is no significant difference [*t*_(11)_ = −1.09, *p* = 0.297]; (2) is there a significant difference in LP_B1 between BPD_patients and controls? As stated previously, there is a significant difference [*t*_(25.04)_ = −2.32, *p* = 0.029], with controls lagging significantly more in section B1 than do BPD patients. In summary, when the music accompaniment changes markedly in section B1, BPD patients do not play significantly less or more in time than do controls, but controls do tend to lag behind the beat more often than do BPD patients. As a final remark in this results section, we point out that in the second stage of a stepwise selection procedure seeded with LP_B1, there are other interesting variables that could make significant improvements to the model (e.g., rhythmic simplicity in section B or RS_B, compression ratio of ontime-duration pairs in sections A and A' or CR_dur_A, CR_dur_A'). These variables did not contribute as significantly as MDB_sd_B1, however, so we did not explore them further, but they could be investigated by the interested reader via the URL given in the caption of Figure 5.

### Additional analysis with the metrical deviation model (MD_m_B1−MD_m_B2)

Based on the significant findings of the metrical deviation model, we calculated a new variable “MD_m_B1−MD_m_B2,” which describes the behavior of improvement in IPS during the B part of the improvisation. The interpretation of this measure is that positive scores on this measure imply a trend in improving IPS behavior. A negative score implies a trend in worsening IPS behavior.

We conducted a series of separate analyses to test possible influences of medication, severity of depression, and musical capacities on IPS behavior. To measure musical capacities, we assessed both perceptual and experiential capacities. Finally, we included a psychological measurement of general attachment, to explore if the variance in “MD_m_B1−MD_m_B2” could be at least partially explained by the underlying fundamental two dimensions of attachment: Avoidance and anxiety.

#### Influence of musical sophistication and beat perception

General musical sophistication, and the additional five subscales (Active Engagement, Perceptual Abilities, Musical Training, Singing Abilities, and Emotions) did not correlate with “MD_m_B1−MD_m_B2” in BPD patients. In normal controls, no significant correlation was found. Neither was beat perception significantly correlated with “MD_m_B1−MD_m_B2” in BPD patients and normal controls.

#### Influence of psychotropic medication and mood in BPD patients

Medication use did not correlate with “MD_m_B1−MD_m_B2” in BPD patients. And also severity of depression did not show a significant correlation.

#### Influence of general attachment style

There was a positive significant correlation between “MD_m_B1−MD_m_B2” and avoidance general attachment style [*r*_(14)_ = 0.68, *p* < 0.01] in BPD patients, and a negative significant correlation between “MD_m_B1−MD_m_B2” and anxious general attachment style [*r*_(14)_ = −0.55, *p* < 0.05].

Avoidance and anxiety dimensions accounted for 52% of the variance in “MD_m_B1−MD_m_B2” in BPD patients [*R*^2^ = 0.5229, *F*_(2, 13)_ = 7.124, *p* < 0.01]. There was no correlation between “MD_m_B1−MD_m_B2” and avoidance and anxiety dimension in normal controls. Finally, we want to make clear that we did not Bonferroni-correct the *p*-values for these correlational analysis. If we do, the remaining significant result concerns avoidant general attachment style.

## Discussion

### Conclusions

With a lifetime prevalence of 5.9% and serious consequences for emotion regulation, impulse control, and interpersonal relationships, BPD is a condition that has been and remains an important subject of research in psychology and neuroscience. Whereas, existing research on BPD—such as self-report questionnaires and unidirectional studies—has not focused on measuring the exchange of social signals between individuals, we attempted to do so via the use of an ABA′ accompanied improvisation paradigm. Impairments in IPS are a known characteristic of BPD, and this paradigm made it possible to measure timing habits in IPS (e.g., the exchange of social signals) over the course of the musical interaction. In the B part of the improvisation (freer improvisation), the intervention from the therapist (accompaniment) invited a greater degree of (social) interaction from the participant. We quantified 15 aspects of each participant's playing across the improvisation sections (A, B, split also into B1and B2, and finally A′), focusing on temporal characteristics that may act as behavioral measures of IPS, as well as some aspects intended to measure impulsivity.

Our main predictions were that there would be: (1) poorer temporal IPS, represented by higher overall timing deviations, for BPD patients compared to normal controls; (2) more oscillations (e.g., more variability) in timing deviations between therapist and BPD patients compared to normal controls; (3) problems in maintaining and improving IPS between therapist and BPD individuals in the course of the joint improvisation compared to normal controls; (4) more impulsivity (less inhibition) in the playing of BPD patients than normal controls. Among our main findings were that: (i) the control group showed significant improvements in IPS over the course of section B2 (variable name MD_m_B2) compared with section B1 (MD_m_B1) of the improvisation, contrary to the BPD group who showed less improvement in IPS over the course of part B (freer improvisation). This finding substantiates prediction 3; (ii) normal controls were significantly more likely to play behind the beat in section B1 (variable name LP_B1) than were BPD individuals, which substantiates prediction 4; (iii) a logistic regression model built on the difference in mean metrical deviation between sections B1 and B2 performed significantly better than chance at categorizing given participants as either having BPD or being a control (82% success rate). So while there was not clear evidence to support predictions 1 and 2 in our findings, we did find evidence to substantiate predictions 3 and 4, as well as a model whose discriminatory power suggests that our behavioral measures of IPS are relevant to the diagnosis of BPD.

#### Overall timing deviations and oscillations in IPS

Contrary to our prediction 1, results showed that difficulties to synchronize with others represented by strong overall timing deviations (as measured by the variable MD_m) was not related to BPD pathology. Neither was evidence of our prediction 2 found in the results—that BPD individuals would show more oscillations in their playing (as measured by the variable MD_sd), such as being very close in time to the therapist followed by tendencies to withdraw from the therapist. This suggests that differences in overall timing deviations and oscillations in temporal IPS in a joint improvisation are not related to BPD characteristics. Probably these specific timing aspects are more related to other individual characteristics as proposed elsewhere (e.g., Loehr and Palmer, 2011). For instance, Loehr and Palmer (2011) address the correlation between individual tempo profiles of two partners (in piano duet performances) and overall timing deviations in temporal IPS in a joint musical interaction. Their study shows that partners who have a similar “tempo profile” synchronize better. Moreover, well-matched partners are better able to simulate the timing of the other (e.g., action simulation), they adapt better to the timing of the other in the course of the interaction, and there is also more mutual adaptation between the two partners, compared to less well-matched partners (Loehr and Palmer, 2011). These findings were also found in research about movement coordination in joint action (Schmidt and Turvey, 1994; Amazeen et al., 1995; Richardson et al., 2007). We suggest that individual differences in tempo profiles between therapist and patients will influence overall timing deviations in a joint musical improvisation instead of BPD characteristics. Further research may gain insight into the influence of therapist/patient tempo profiles in therapeutic processes.

#### Maintaining and improving temporal IPS

As expected, results showed that BPD patients had difficulties in maintaining and improving temporal IPS during the improvisation compared to normal controls. This was only visible within the B part (in particular, the B1–B2 transition) of the improvisation, where the therapist's playing invited more musical interaction compared to parts A or A', where the therapist was repeating a short, stable pattern. In other words, when the (insecure) attachment system of the patient was activated, difficulties were found in maintaining and improving temporal IPS in musical improvisations with BPD patients.

In addition, we suppose that the underlying cognitive motor skills associated with anticipation (Keller et al., 2007; Pecenka and Keller, 2009; Rankin et al., 2009) and adaptation (Large and Jones, 1999; Repp, 2001, 2011; Large et al., 2002; Repp and Keller, 2008; Loehr et al., 2011; Repp and Su, 2013) are hindered in their ability to regulate and facilitate improvements in temporal IPS when the attachment system is activated in BPD patients.

Taken together, it could be that inner representations of attachment relationships and/or the quality of such relationships are embedded/embodied in cognitive-motor strategies of BPD patients, and that anticipatory mechanisms related to prediction errors are hindered in their capacity to maximize prediction of the future. Brain reward mechanisms are known to regulate prediction errors. In this sense, our findings seem to support current theories about the relation between alterations in the brain reward system in BPD individuals, attachment and prediction error (Friston, 2005, 2010; Atzil et al., 2011; Fonagy et al., 2011; Brown and Brüne, 2012; Enzi et al., 2013; Herpertz and Bertsch, 2015).

Our findings may have interesting implications in relation to music-therapeutic embodied strategies. If, within the music-therapeutic process, BPD patients can experience repeated experiences of “good enough” temporal IPS, this could lead to implicit repair of maladaptive embodied timing strategies, related to attachment experiences. This might mitigate affectively-oriented interpersonal features in BPD patients, such as intolerance of loneliness, conflicts with dependency, discomfort with care, and fear of abandonment. These suggestions are consistent with research suggesting that attachment patterns could be changed as a result of significant changes in relationships (e.g., Waters et al., 2000). However, we have to be careful about making such predictions, because the findings in our study are based on a cross-sectional experiment and thus are not related to longer and more complicated therapeutic interpersonal processes.

In a recent study (Choi-Kain et al., 2010) an important distinction was made between core affectively-oriented interpersonal features (e.g., attachment fears, intolerance of loneliness) and behavioral interpersonal features (e.g., sadism, entitlement, boundary violations, recurrent breakups, demandingness). In particular it was shown that the core affectively-oriented interpersonal features are more persistent than behavioral interpersonal features. The affectively-oriented symptoms are slower in remission and 15–25% of people with BPD did not show improvement in these symptoms compared to baseline in a 10-year follow-up (Choi-Kain et al., 2010). Our findings promote music therapy as a possible complementary therapy in the current field of evidence-based treatments, especially for treating these affectively oriented interpersonal problems, such as attachment fears, with BPD patients. That said, longitudinal research is necessary to put these hypotheses to the test.

Finally, our findings may further augment the expertise and knowledge of music therapists, offering new tools with which to attune to the timing capacities of the BPD patient, with the aim of making improvements more readily, or where none could be made before.

#### Impulsivity and temporal IPS

Results showed that normal controls have the tendency to play their notes significantly more often behind the notes of the therapist than did the BPD patients (so-called lag proportion) in section B1 of the improvisation. This is consistent with our assumption that normal healthy controls are more inhibited in their timing than BPD patients. This is only visible in B1—in the first part of the freer improvisation. When the interaction comes to the fore (part B), BPD patients seems less inhibited, and seem to pursue the more immediate reward of joining the interaction. This is in accordance with previous research about the specificity of impulsivity in BPD patients. In a recent study concerning impulsivity in BPD individuals, a distinction was made between (a) choice or reward-related impulsivity and (b) motor impulsivity (Barker et al., 2015). In particular, the results showed that motor impulsivity was not significantly different between BPD individuals and controls, instead reward-related impulsivity was significantly greater in BPD individuals. Reward-related impulsivity is characterized by choices of small immediate reward, with a focus on the present and with little regard to the future.

Our results suggest that BPD patient have the tendency to pursue the musical interaction more immediately (reward-related impulsivity), relative to healthy controls, who have the tendency to wait longer to join the interaction.

It is plausible that the impulsive playing behavior in BPD patients interferes with our previously described finding about attachment-related impaired maintenance of IPS in joint improvisation. A structural relationship between adult attachment style and impulsivity trait is described in different opposing theoretical models (Scott et al., 2009). The two most common models are: (1) when the insecure attachment system is activated, one cannot rely anymore on secure, adaptive and support-seeking coping. Deficiences in coping strategies may intensify central traits such as impulsivity in BPD patients (Levy et al., 2006). In our study this means that because of the activation of the insecure attachment sytem in the B part of the improvisation, the impulsive behavior in BPD patients is intensified, as seen in B1; (2) an opposing theoretical model contends that the dispositional trait of impulsivity can impede joint interactions, and may contribute to disturbed attachment styles (e.g., Eisenberg et al., 1997, 2000). In our study this means that because of the impulsive behavior in B1, there are difficulties in improving IPS in the course of the improvisation. In this sense, the impulsive behavior impedes the joint interaction in BPD patients. We have to be cautious with possible interpretations, however, because our study was not designed to reveal causal relationships. In either case, our findings are consistent with the existence of a relationship between impulsivity traits and attachment difficulties with regard to impairments in temporal IPS.

#### Ecological validity

We would like to stress the ecological validity of this study—that a free musical improvisation approximates social collaboration in the real world more than do experimental studies that make use of methods such as virtual partners (e.g., Repp, 2005; Fairhurst et al., 2013, 2014). It is our premise that the complex and intensive interactions arising from a (freer) musical improvisation are more likely to activate the (insecure) attachment system in BPD patients.

### Limitations

This study has some limitations. First our sample is too small to claim any generalizability of our findings. Second, the individuals with BPD in our sample group participated in the context of an inpatient treatment facility, so our results may not be generalizable to other BPD patients. Further research in bigger and other samples is needed. Third, the beat tracking in this study was done manually, which may cause subjective interferences. Apart from using a beat-tracking algorithm (which may be error prone and so require manual, subjective corrections anyway), we suggest synchronization tasks with computer-generated pacing signals as a possible means of reducing subjectivity. While such tasks miss the human deviations (i.e., variations in timbre and intensity) in the joint improvisation, studies on action simulation have demonstrated that even very reduced stimuli can be experienced as a human product with social meaning (Steinbeis and Koelsch, 2009; Sevdalis and Keller, 2011). Finally, we included only the subscale of the general attachment style in this study. In future research, the assessment of attachment style across several distinct relationships is necessary to gain better insights into attachment-related correlations.

### Future directions

In terms of assessment and diagnosis of BPD, the methods developed in our study could become part of a comparatively lightweight tool to detect possible cases of BPD, in order to reduce the need to administer more onerous questionnaires. With regards treatment of BPD, longitudinal research (such as randomized controlled trials) in music therapy is needed to investigate the extent to which improvement in implicit interpersonal processes of IPS is correlated with the improvement of affectively oriented interpersonal functioning in BPD. In terms of both assessment and treatment, recent improvements in machine learning techniques and musical improvisation invite the possibility that accompaniments for participants could be computer-generated in real time, and thus musical parameters of the accompanist could be controlled more exhaustively, which might lead to more objective measures of (the development of) a participant or patient's musical behavior.

## Author contributions

KF and JDB designed and performed experiments; KF analyzed data and wrote the paper; KF and TC developed MIR variables; TC wrote code and ran the analysis; KF and TC prepared the figures; KF, JDB, and TC discussed the results; TC and JDB edited the paper.

## Funding

Funding source, OPaK (LUCA, School of Arts, KULeuven).

### Conflict of interest statement

The authors declare that the research was conducted in the absence of any commercial or financial relationships that could be construed as a potential conflict of interest.

## Footnotes

^1^The data, analyses, and plots that underpin the paper are available at http://bit.ly/2bgT77f.

^2^Selection proceeded from the null model according to improvement (reduction) in Aikaike's information criterion (AIC). It was not possible for selection to proceed from a full model because we had more independent variables than data points.
