# Supplementary material for: Impaired Maintenance of Interpersonal Synchronization in Musical Improvisations of Patients with Borderline Personality Disorder
Source: Front Psychol. 2017 Apr 27;8:537. doi: 10.3389/fpsyg.2017.00537 (PMC5407194; doi:10.3389/fpsyg.2017.00537)
Supplement: Supplementary file 1 [file DataSheet1.DOCX]

Supplementary Material

Impaired maintenance of interpersonal synchronization in musical improvisations of patients with borderline personality disorder

**Katrien Foubert^*^, Tom Collins, Jos De Backer**

*** Correspondence:** Katrien Foubert: katrien.foubert@luca-arts.be

## Supplementary details of music data processing and mathematical definitions of MIR variables

Pre-processing of the music data began with beat-tracking the MIDI files of the improvisations. This task was undertaken by a professional musician/music therapist using the Logic Pro X software (see Supplementary Figure 1). The beat-tracking was done based on the accompanist’s playing (the participant’s part was muted). We explored the possibility of using automatic beat-tracking technology, but determined it would take longer to train, run, and correct the system than it would to beat-track the files manually in the first place. In the first round of beat-tracking, the professional musician tapped the beat into Logic while the music was playing, and then went back through the files several times to correct any inaccuracies. The MIDI improvisation and beat-track files were then imported into the Lisp package MCStylistic and Matlab package PattDisc [(Collins, 2011)](https://paperpile.com/c/sng0b2/NHEyP) in order to produce a quantized version of the improvisation (each performed note was mapped to a start time commensurate with how it would be written in staff notation). Listening to the accompanied improvisations, it was clear that in participants’ playing, eighth notes were the most common subdivision of the therapist’s beat. As such, quantization was done automatically on this basis.


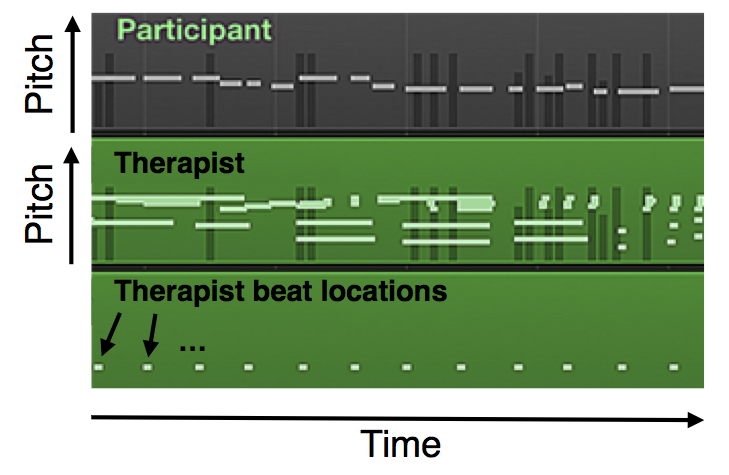


**Supplementary Figure 1.** Screenshot from Logic, showing the channels for participant, therapist, and beat track of the therapist’s accompaniment. Time runs from left to right, and the somewhat compressed vertical axis indicates what pitches are being played when and for how long (light horizontal oblongs). Darker vertical oblongs indicate the foot pedal presses of the therapist, but do not form part of our analysis.

**IPS**

Several variables were calculated, all based on deviations between the onsets of a participant and those of the eighth-note subdivisions of the therapist’s beat in a given section, as indicated in Figure 3 of the main article.

(1) MD_m stands for mean metrical deviation. Each note onset *y_i_* performed by a participant (a time in seconds) can be linked to the underlying eighth-note beat to which it is closest, and the time *x_i_* in seconds at which this beat occurs. If a participant plays *n* notes, then MD_m is the mean of the absolute deviations between each played note onset and the time of the closest underlying beat: MD_m = (1/*n*)Σ*_i_* _= 1,...,_ *_n_*|*y_i_* − *x_i_*|. The variable MD_m is always non-negative. The larger the value of MD_m, the more the participant deviates from the beat over an improvisation section, and the more ‘out of time’ their playing will sound. We use this as one operational definition for the participant’s level of IPS. This variable says nothing about whether the participant is consistently out of time, however, or whether (s)he tends to play ahead of or behind the beat.

(2) MD_sd  is the standard deviation of the metrical deviations |*y*_1_ − *x*_1_|, |*y*_2_ − *x*_2_|,..., |*y_n_* − *x_n_*|. The variable MD_sd is always non-negative. If a participant plays some notes out of time but most in time (as in Figure 3), (s)he will have a larger value of MD_sd than a participant who is consistently out of or in time. We use MD_sd as an operational definition for the consistency of a participant’s IPS.

(3) LP stands for lag proportion, and is the proportion of times that a participant’s notes are behind the beat over an improvisation section. If we let *I*(*y_i_* − *x_i_* > 0) be an indicator function for the positivity of *y_i_* − *x_i_*, where *y_i_* is a note onset performed by a participant and *x_i_* is the time of the beat to which it is closest, then the indicator function takes the value 1 when the participant plays behind (after) the beat and 0 otherwise. Therefore, LP = (1/*n*)Σ*_i_* _= 1,...,_ *_n_ I*(*y_i_* − *x_i_* > 0) is the proportion of times that a participant’s notes are behind the beat. If a participant is always ahead of the beat, then LP = 0; if a participant is always behind the beat, then LP = 1. As the mean metrical deviation MD_m does not indicate whether a participant tends to play ahead of or behind the beat, this is the potential contribution of a variable such as lag proportion LP. A possible issue with LP is that it does not measure the *extent* to which a participant plays ahead of or behind the beat, only the *tendency* to be ahead or behind.

(4) MDA_m is the mean of the metrical deviations *y_i_*_(1)_ − *x_i_*_(1)_, *y_i_*_(2)_ − *x_i_*_(2)_,..., *y_i_*_(_*_m_*_)_ − *x_i_*_(_*_m_*_)_ that are ahead of the beat, where { *i*(1), *i*(2),..., *i*(*m*) } is the subset of the indices { 1, 2,..., *n* } for which *y_i_* − *x_i_* < 0. MDA_m is always negative. This variable captures the extent to which a participant plays ahead of the beat.

(5) MDA_sd is the standard deviation of the above. If a participant plays some notes a long time before the beat but most just before, (s)he will have a larger value of MDA_sd than a participant who plays fairly consistently before the beat.

(6) MDB_m is the mean of the deviations that are behind the beat. It is complementary to MDA_m.

(7) MDB_sd is the standard deviation of the above. It is complementary to MDB_sd.

We use the variables LP, MDA_m, MDA_sd, MDB_M, MDB_sd as proxies for the directionality of a participant’s IPS – the extent to which (s)he is ahead of or behind the accompanist.

**Tempo**

Tempo and variations in tempo were measured based on the inter-onset interval (IOI) of the beat track. IOI is the difference between two consecutive quarter-note beats. Tempo in beats per minute and IOI are related by the formula tempo = 60/IOI, so the smaller the IOI, the faster the tempo.

(8) TMP_m stands for mean tempo. Letting *x*_1_, *x*_2_,..., *x_n_* be the tempi between each pair of consecutive quarter-note beats, TMP_m = (1/*n*)Σ*_i_* _= 1,...,_ *_n_ x_i_*. TMP_m is always positive. If an improvisation section sounds fast, then likely the mean tempo for that section is large, compared to a section that sounds slow. We use this as an operational definition of the participant’s propelling (or, on the other hand, holding back the progress) of the improvisation.

(9) TMP_sd stands for tempo standard deviation. It captures the variability in the tempo over an improvisation section.

**Rhythmic motifs/patterns**

Rhythmic motifs or patterns were quantified using the quantized improvisation data.

(10) CR_dur is the compression ratio applied to transcribed (ontime, duration)-pairs. For instance, in Figure 5C of the main article, a participant plays the transcribed (ontime, duration)-pairs

{ (0, 1), (1, 1), (4, 1),..., (30, 1), (32, 1), (33, 1), (36, 1),..., (62, 1), (64, 1), (65, 1), (4, 1),..., (94, 1) },

if we expand the two repetitions. We know there is repetition in this set, and so these 57 (ontime, duration)-pairs can be represented as a set *S*_1_ = { (0, 1), (1, 1), (4, 1),..., (30, 1) } of nineteen pairs and two translation vectors *t*_1_ = (32, 0), *t*_2_ = (64, 0) that bring *S*_1_ to the two subsequent occurrences. In this way, the set of 57 pairs can be represented more concisely with 21 pieces of information – nineteen members of *S*_1_, plus the two translation vectors. We talk about a *compression ratio* of 2.71 ≅ 57/21. These so-called *translational patterns* in (ontime, duration)-point sets can be discovered automatically using a pattern discovery algorithm. We used the SIARCT-CFP algorithm (Collins et al., 2016), which will also handle certain categories of inexact repetition or variation. The more rhythmic motifs or patterns in a participant’s playing, the more their corresponding (ontime, duration)-point set tends to be compressible, and the higher their compression ratio will be.

(11) TC_o is the translational coefficient applied to transcribed ontimes. It measures the degree of randomness in an ontime set with 0 for least random and 1 for most random. According to this variable, the least random set of *n* ontimes is one where the members are isochronous, *S* = { *j* + *k*, 2*j* + *k*,..., (*n* − 1)*j* + *k* }, where *j*, *k* ∈ R. If we calculate the differences between each pair of members of *S*, there are only *n* − 1 unique values: the difference between consecutive members of *S* is always *j*; the difference between consecutive-but-one members of *S* is always 2*j*;...; the difference between the first and last members is (*n* − 1)*j*. At the other extreme, it is possible to observe or define a maximally random set *S* of *n* ontimes such that no two differences between pairs of members are the same, and in this case there are *n*(*n* − 1)/2 differences. For instance, the set *S* = { 1, 2, 4, 8 } of *n* = 4 ontimes is such that no two differences between pairs of members are the same: the differences are 1, 2, 4, 3, 6, 7 and in this case there are 6 = 4(4 − 1)/2 = *n*(*n* − 1)/2 of them. For a given set of *n* ontimes, we can calculate the number *M* of unique differences between pairs of members and then map this number linearly to [0, 1], using the two extreme cases just described for the normalization, which gives the translational coefficient TC_o = [*M* − (*n* − 1)]/[*n*(*n* − 1)/2 − (*n* − 1)], simplifying to TC_o = 2[*M* − *n* + 1]/[(*n* − 1)(*n* − 2)], for *n* > 2. Similar to compression ration CR_dur, the translational coefficient TC_o should measure the extent to which a participant’s rhythmic playing is random. The benefit of the latter over the former is that TC_o is parameter-free.

(12) RS stands for rhythmic simplicity, and is defined as the proportion of inter-ontime intervals belonging to the modal bin in an inter-ontime histogram. We used bin sizes of eighth notes. If a quantised improvisation is ‘rhythmically simple’, most of its inter-ontime intervals belong to the modal bin, and so the proportion belonging to this bin – what we term rhythmic simplicity – will be close to 1.

**Interpersonal imitation**

The final three variables aim to capture ways in which the participant may have recognized and imitated aspects of the accompanist’s playing.

(13) DN stands for note density, and is given by the number of notes played over an improvisation section, divided by the length of the section in seconds. The note density of the accompanist was much lower in sections A and A’ than it was in section B, for example, so if this was recognized and imitated by participants, the note density variable has the potential to capture it.

(14) AI_mu is the mean articulation interaction, attempting to measure the extent to which the participant imitates durations in the accompanist’s playing. We calculated this by comparing note durations played in windows of length just over one bar, just over every half bar, at a lag of just over one quarter-note beat between accompanist and participant. If the accompanist played durations *z*_1_, *z*_2_,..., *z_m_* in window *k*, and the participant played durations *w*_1_, *w*_2_,..., *w_n_* in the corresponding lagged window, then the absolute difference between the durations means, *a_k_* = |(1/*n*)Σ*_i_* _= 1,...,_ *_n_ w_i_* − (1/*m*)Σ*_j_* _= 1,...,_ *_m_ z_j_*|, gives the instantaneous articulation interaction, where *m*, *n* > 0. To get the mean articulation interaction, we calculate AI_mu = (1/*K*)Σ*_k_* _= 1,...,_ *_K_ a_k_*. The lower the value of AI_mu, the closer a participant’s durations are to those of the therapist, and we take this as a proxy of imitation.

(15) AI_min is the minimum articulation interaction. AI_min = min{ *a_k_* | *k* = 1, 2,..., *K* }. We include this variable because a few similar durations between participant and therapist may give rise to the impression of imitation, even if other durations deviate more. The minimum function may capture such instances whereas a mean may not.

## Supplementary Results

Supplementary Figure 2 shows the distribution for each of our MIR variables, split according to patients and controls, as well as according to the different sections of the accompaniment. In temporal order, these sections are A, B, Aʹ, but for the sake of inspecting the results, it makes sense to place A and Aʹ next to one another (columns 1 and 2), and to place B (column 3) next to its bisection into B1 and B2 (columns 4 and 5). For example, in the third row, fifth column of Supplementary Figure 2 is the plot for the lag proportion variable in part B1. It has a darker background to indicate that the difference between patients and controls for this variable is significant. No other variables showed significant differences when considered in isolation.

**Supplementary Figure 2.** Violin plots of each of the fifteen MIR variables, split on the *x*-axes by patient (= 1) and control (= 0). Variable values are shown as *z*-scores for ease of presentation. Each row shows a different variable and each column shows its distribution for the different sections of the accompaniment (A, Aʹ, B, B1, and B2). The darker background for variable LP in section B1 indicates the difference between patients and controls is significant at the .05 level.
